# Three live-imaging techniques for comprehensively understanding the initial trigger for insulin-responsive intracellular GLUT4 trafficking

**DOI:** 10.1016/j.isci.2022.104164

**Published:** 2022-03-26

**Authors:** Hiroyasu Hatakeyama, Ko Kobayashi, Makoto Kanzaki

**Affiliations:** 1 Frontier Research Institute for Interdisciplinary Sciences, Tohoku University, Sendai 980-8579, Japan; 2 Graduate School of Biomedical Engineering, Tohoku University, Sendai 980-8579, Japan; 3 Department of Physiology, Kitasato University School of Medicine, Sagamihara 252-0374, Japan

**Keywords:** Optical imaging, Biological sciences, Cell biology, Biological sciences research methodologies, Biology experimental methods

## Abstract

Quantitative features of GLUT4 glucose transporter’s behavior deep inside cells remain largely unknown. Our previous analyses with live-cell imaging of intracellular GLUT4 trafficking demonstrated two crucial early events responsible for triggering insulin-responsive translocation processes, namely, heterotypic fusion and liberation. To quantify the regulation, interrelationships, and dynamics of the initial events more accurately and comprehensively, we herein applied three analyses, each based on our distinct dual-color live-cell imaging approaches. With these approaches, heterotypic fusion was found to be the first trigger for insulin-responsive GLUT4 redistributions, preceding liberation, and to be critically regulated by Akt substrate of 160 kDa (AS160) and actin dynamics. In addition, demonstrating the subcellular regional dependence of GLUT4 dynamics revealed that liberated GLUT4 molecules are promptly incorporated into the trafficking itinerary of transferrin receptors. Our approaches highlight the physiological significance of endosomal “GLUT4 molecule trafficking” rather than “GLUT4 vesicle delivery” to the plasma membrane in response to insulin.

## Highlights

• Insulin triggers heterotypic fusion of static GLUT4-vesicles with traveling endosomes
• GLUT4 molecules liberated into the fused endosome expropriate its vital traffic path
• Actin dynamics and AS160 are essential for insulin-dependent heterotypic fusion event
• Key role of GLUT4 “molecule trafficking” upon heterotypic endosomal fusion

## Graphical abstract

**Figure:**
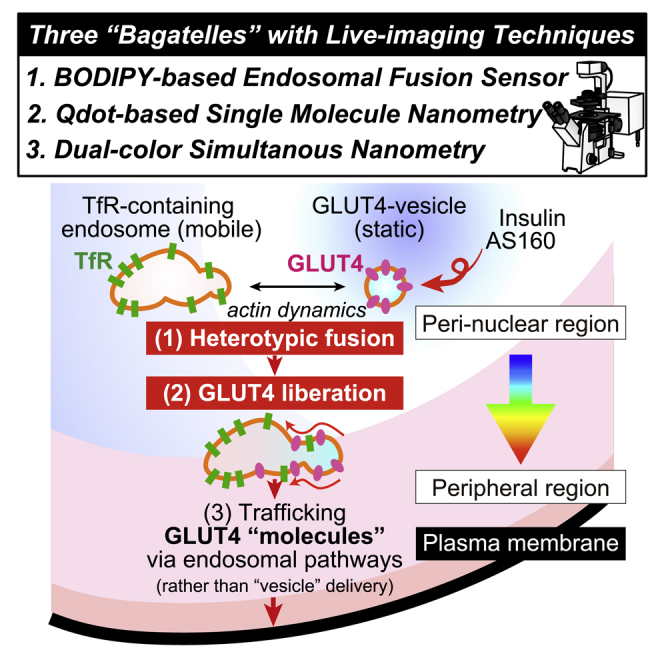

## Introduction

The insulin-responsive glucose transporter GLUT4 mediates insulin-dependent facilitation of glucose uptake in tissues displaying the highest levels of insulin-dependent glucose uptake such as adipocytes and skeletal muscle (Cushman and Wardzala, 1980; Suzuki and Kono, 1980). GLUT4 undergoes stimulus-dependent trafficking between intracellular pools and the cell surface, i.e., insulin facilitates GLUT4 translocation from a specialized intracellular pool called the GLUT4 storage compartment to the plasma membrane via activation of the intracellular signaling cascade including phosphatidylinositol (PI) 3-kinase, Akt, and AS160 (Saltiel and Kahn, 2001; Watson et al., 2004). Because dysfunction of these processes is directly related to type 2 diabetes development, detailed quantitative understanding of these processes, which provide essential keys to glucose homeostasis and are involved in the etiology of type 2 diabetes, is necessary. Indeed, GLUT4 is now one of the best-studied trafficking proteins, and the qualitative features of GLUT4 trafficking processes have also been extensively investigated. However, even for such a well-studied protein, quantitative features of intracellular GLUT4 trafficking, especially events occurring deep inside cells, where the key regulatory sites of the entire process reside, are as yet poorly understood as compared to the qualitative features of the processes involved. We previously analyzed intracellular GLUT4 trafficking properties with our own live-cell imaging approaches including high precision measurements of intracellular movements with single-molecule imaging of Quantum dot (QD) fluorescent nanocrystals and a cell biological technique employing a fluorescent sensor molecule (Fujita et al., 2010; Gardini et al., 2018; Hatakeyama and Kanzaki, 2011, 2013a, 2013b, 2017; Hatakeyama et al., 2019; Hatakeyama et al., 2017). By focusing on GLUT4 and the transferrin receptor (TfR), a general recycling protein that does not show as much insulin-responsive translocation as GLUT4, we have obtained quantitative insights into GLUT4 trafficking properties with these approaches. For example, statistical comparison of GLUT4 and TfR behaviors clearly documented a unique regulatory system for GLUT4. Specifically, we identified putative anchoring mechanisms statically retaining GLUT4 in their storage compartments, especially around the perinuclear trans-Golgi network region, and insulin-responsive “liberation” from this “static” GLUT4 anchoring system in 3T3L1 adipocytes (Hatakeyama and Kanzaki, 2011) and skeletal myofibers (Hatakeyama and Kanzaki, 2017). Because insulin-responsive liberation is a prerequisite for GLUT4 reaching the plasma membrane, the unique regulatory system identified here is the most important initial element of insulin action regulating the entire GLUT4 trafficking itinerary. We also identified acute “heterotypic endosomal fusion” of very small static GLUT4-containing vesicles (GLUT4-vesicles) with a subset of TfR-containing endosomes as another critical initial process in the insulin-responsive GLUT4 translocation, in addition to insulin-responsive liberation, in mouse skeletal myofibers (Hatakeyama and Kanzaki, 2017). These two distinct principle-based imaging techniques, focused on elucidating the same biological phenomena, raise simple questions regarding the relationship between GLUT4 liberation and GLUT4-vesicle fusion, as well as their subcellular regional dependence. To directly answer the questions raised, experimental approaches allowing more rigorous quantification than those we previously developed are necessary. Therefore, we herein performed three distinct analyses, each based on our dual-color live-cell imaging technique, to further precisely and comprehensively quantify and display the crucial initial events of insulin-responsive GLUT4 trafficking, i.e., 1) dual-color imaging-based ratiometric analyses of heterotypic fusion aimed at elucidating the regulatory processes with rigorous quantification, 2) dual-color imaging of heterotypic fusion and individual GLUT4 movements for clarifying the interrelationships between heterotypic fusion and liberation, and 3) dual-color single-molecule imaging for evaluating intracellular trafficking properties and the subcellular regional dependence of their dynamics.

## Results

### Technique 1: Ratiometric analysis of insulin-responsive heterotypic fusion in 3T3L1 cells

**Figure 1:**
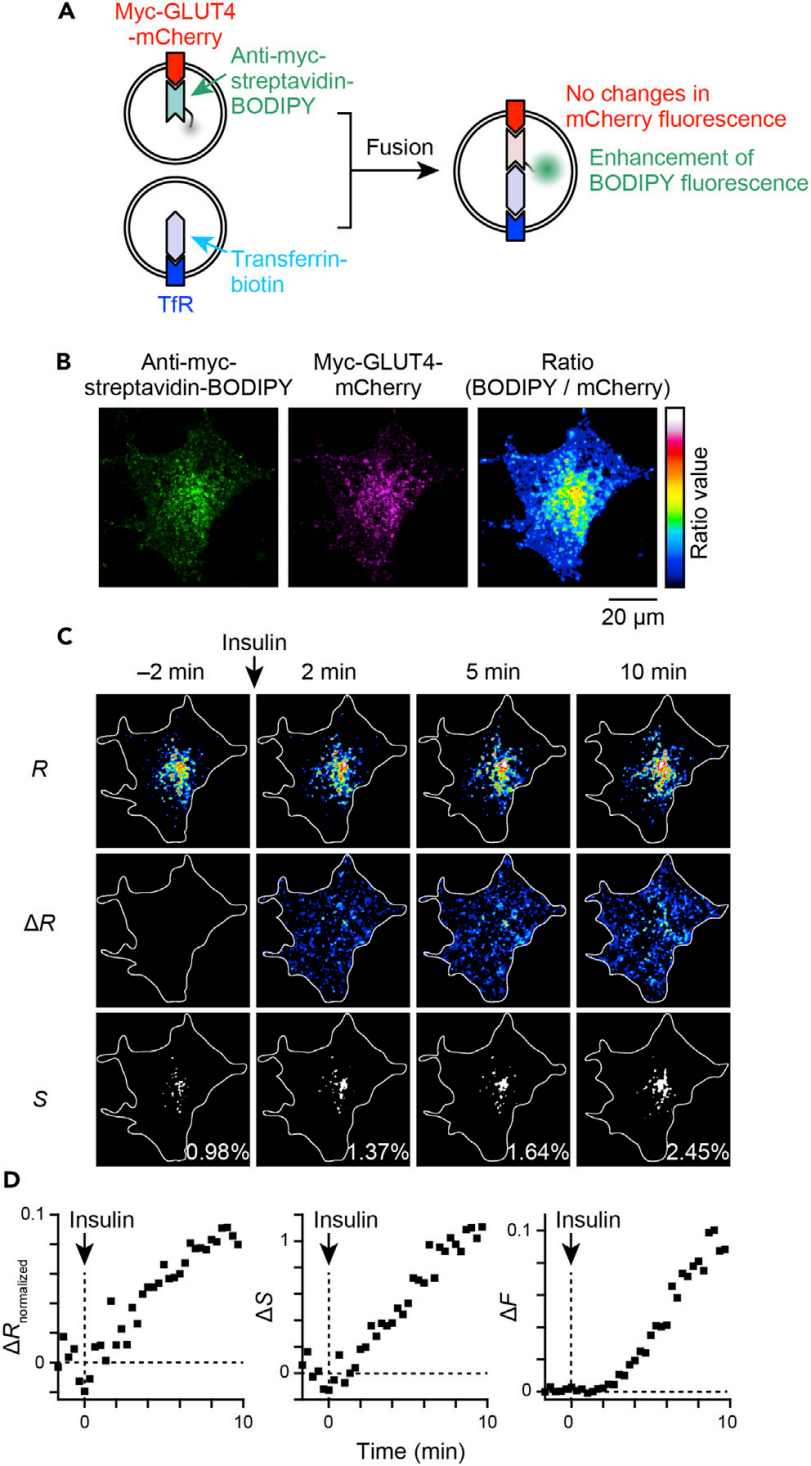
Schema for quantifying heterotypic endomembrane fusion with ratiometric analysis (A) Improved fusion experiments with dual-color imaging. Before stimulation, myc-GLUT4-mCherry and TfR were labeled with BODIPY/streptavidin-conjugated anti-myc antibodies and transferrin-biotin, respectively. After myc-GLUT4-mCherry–containing vesicles fuse with TfR-containing endosomes, biotin-streptavidin binding dequenches BODIPY fluorescence, such that increases in green fluorescence can be observed without changes in mCherry fluorescence. (B) Fluorescent images of BODIPY/streptavidin-conjugated anti-myc antibodies (*left*) and myc-GLUT4-mCherry (*center*), and the ratio image obtained by dividing BODIPY fluorescence by mCherry fluorescence (*right*) in a 3T3L1 adipocyte expressing myc-GLUT4-mCherry and TfR. Cells were first labeled with BODIPY/streptavidin-conjugated anti-myc antibodies for 1 h, washed for at least 3 h, and then treated with 5 μg/mL of transferrin-biotin for 5 min. Images were acquired after 10 min of washout of transferrin-biotin. The ratio image was median filtered. A color table showing the ratio value is also presented. (C) Typical changes in the fluorescence ratio in response to insulin stimulation (100 nM) at time 0 in the cell are shown in (B). Ratio images (*R*, *upper*), images of changes in the ratio values that were calculated by *R*–*R*_0_, where *R*_0_ is the mean ratio value of the cellular region (white lines) for the frames imaged during the 2 min period before insulin stimulation (Δ*R*, *middle*), and binary images showing the area for which the ratio value is above the threshold that was set as the top 1% ratio value within the cellular region (*S*, *bottom*) are shown. In the Δ*R* images, negative values are not shown. In the *S* images, percent areas within cellular regions are also shown. (D) Quantification of insulin-responsive heterotypic endomembrane fusion calculated from the cell shown in (C). Time-dependent changes in the Δ*R*_normalized_, which is the normalized value of Δ*R* with *R*_0_ (Δ*R*_normalized_, *left*), Δ*S* (*middle*), and their multiplication (Δ*F*, *right*) are presented. See also Figures S1–S3.

First, we sought to determine whether insulin-responsive heterotypic endosomal fusion of GLUT4 vesicles with TfR-containing endosomes occurs in 3T3L1 cells, one of the cell lines for which GLUT4 translocation processes have been extensively analyzed, as observed in skeletal myofibers. In our previous study, demonstrating acute heterotypic endosomal fusion, we measured changes in the fluorescent intensity of the fusion sensor molecule BODIPY/streptavidin-conjugated anti-myc antibodies in skeletal myofibers isolated from myc-GLUT4-EGFP-transgenic mice (Hatakeyama and Kanzaki, 2017). This analysis was based on enhancement (dequenching) of the fluorescence of BODIPY, which is quenched by energy transfer from streptavidin via binding to biotin (Figure S1A) (Emans et al., 1995). In 3T3L1 adipocytes, insulin can enhance BODIPY fluorescence as in skeletal myofibers, and this enhancement was observed only in the presence of TfR labeling with Tf-biotin (Figure S1B), suggesting that insulin can also induce heterotypic fusion in 3T3L1 adipocytes. However, in our prior studies, changes in BODIPY fluorescence were traced only within arbitrarily set regions of interest, and the subcellular regional dependence of heterotypic fusion has not been determined. Importantly, the fluorescent intensities could be altered because of factors other than fusion reactions, such as movements of the fluorescent molecules themselves and focus drift. These are non-negligible problems encountered when endeavoring to accurately examine heterotypic fusion in all cell regions. To overcome this drawback and to achieve accurate measurement, ratiometric analysis employing two distinct fluorophores — one whose intensity changes and the other whose intensity does not change during heterotypic fusion — are anticipated to be effective. Herein, we utilized 3T3L1 cells expressing myc-GLUT4-mCherry and first labeled the cells with BODIPY/streptavidin-conjugated anti-myc antibodies in the presence of 1 nM insulin. The labeled molecules were then allowed to recycle back to their stationary compartments, followed by TfR labeling with biotinylated transferrin (Hatakeyama and Kanzaki, 2017). When heterotypic fusion occurs between the two vesicles, the BODIPY fluorescence is expected to be enhanced, whereas mCherry fluorescence remains unchanged (Figure 1A). We traced the fluorescence intensities of BODIPY and mCherry and found obvious increases in BODIPY but not mCherry fluorescence in response to insulin (Figures S2A and S2B). In this example, mCherry fluorescence was somewhat decreased after insulin stimulation (Figure S2B), reflecting factors other than heterotypic fusion. Therefore, we calculated the BODIPY/mCherry fluorescence ratio (Figure 1B) and observed its enhancement upon insulin stimulation (Figures 1C and S2C). Intraluminal pH changes along the endosomal pathway, but the fluorescence of BODIPY and mCherry as well as biotin-streptavidin binding are known to be resistant to pH changes over a wide range (Chaiet and Wolf, 1964; Shaner et al., 2004; Urano et al., 2009). The dequenching property of streptavidin-conjugated BODIPY by biotin binding is also reportedly insensitive to changes in pH (Emans et al., 1995). Therefore, enhancement of the BODIPY/mCherry fluorescence ratio reflects the occurrence of heterotypic fusion but not pH changes along the endosomal pathway. In addition, high local concentrations of BODIPY can cause prominent self-quenching of the dye (Liu et al., 2019), and it may therefore be possible that a high packing density of labeled myc-GLUT4 within the storage compartments results in self-quenching of BODIPY that can dequench in response to insulin-dependent liberation. Indeed, myc-GLUT4 labeling in the presence of 100 nM insulin instead of the 1 nM resulted in fainter BODIPY fluorescence, although it presumably increased the amount of labeled myc-GLUT4 molecules (data not shown). This may be at least in part because of the self-quenching property of BODIPY. However, considering that there were no increases in BODIPY fluorescence with insulin stimulation in the cells labeled without Tf-biotin, as mentioned before (Figure S1B), insulin-responsive increases in BODIPY fluorescence would appear to be derived from heterotypic fusion rather than being attributable to lack of self-quenching of the dye. We also found that brief (5 min) insulin stimulation increased co-localization of the labeled myc-GLUT4 and TfR by about 11% (Figure S3), as in skeletal myofibers. Insulin increased the absolute value of the ratio (*R*, Figure 1C, *upper*: changes in the ratio from before insulin stimulation (Δ*R*) are also shown in the *middle*) as well as the area of which the ratio value was above a certain threshold within a cell (*S*, Figure 1C, *lower*). Therefore, we first quantified the ratio image with two values; one was the normalized changes in the ratio value within the cellular regions (Δ*R*_normalized_, Figure 1D, *left*), and the other was the changes in the area of which the ratio value was above the threshold, set as the top 1% ratio value within the cellular region (Δ*S*, Figure 1D, *center*). Then, we defined the index of insulin-responsive heterotypic fusion (Δ*F*) as the product obtained by multiplying these two values (Figure 1D, *right*).

**Figure 2:**
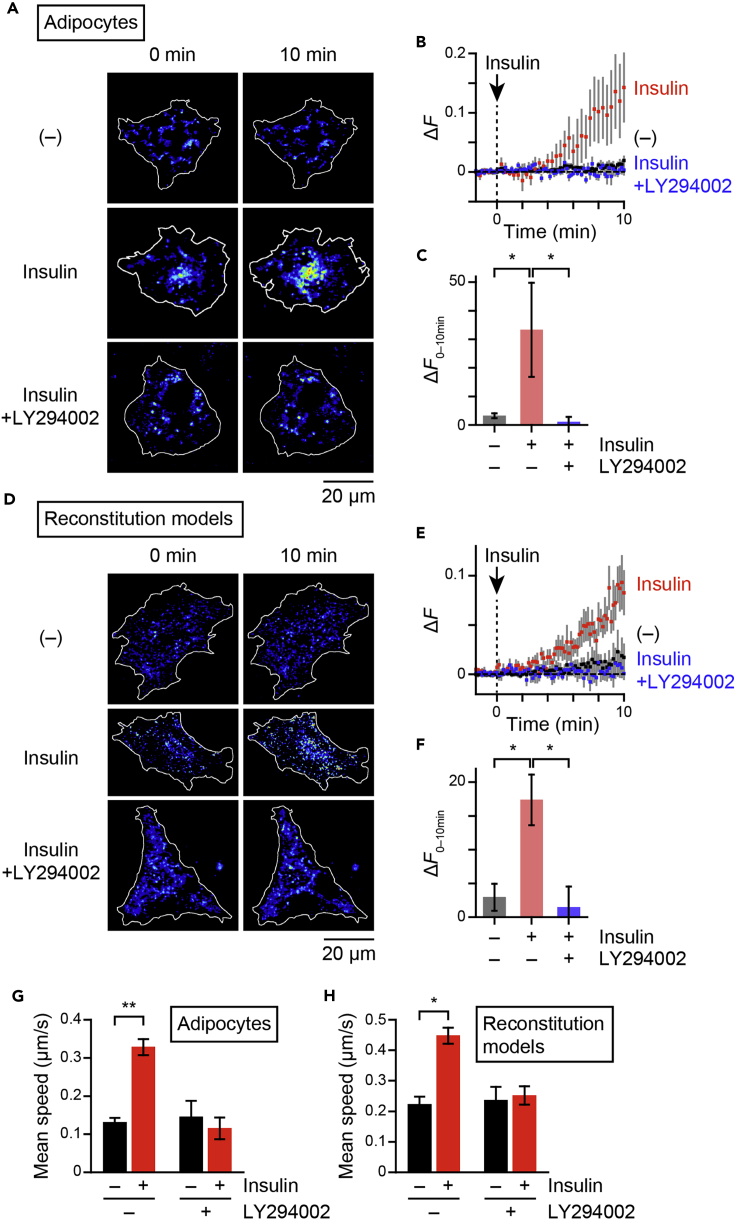
PI 3-kinase-dependent heterotypic endosomal fusion and GLUT4 movement in 3T3L1 cells (A–F) Representative images of Δ*R* before and after insulin stimulation (A and D), changes in Δ*F* (B and E), and areas-under-the-curve of Δ*F* during the 10-min period after insulin stimulation (Δ*F*_0–10min_) (C and F) in either 3T3L1 adipocytes expressing myc-GLUT4-mCherry and TfR (A–C) or the reconstitution models (i.e., 3T3L1 fibroblasts expressing myc-GLUT4-mCherry, HA-sortilin, and HaloTag-AS160) (D–F) under the indicated conditions (*n* = 4–8 cells). Cells that had been labeled with BODIPY/streptavidin-conjugated anti-myc antibodies were first treated with 5 μg/mL of transferrin-biotin for 5 min, washed for 10 min, followed by stimulation with (*red*) or without (*blue*) insulin (100 nM) at time 0. Some cells were pretreated with LY294002 (50 μM) for 10 min before stimulation with insulin (*black*). ∗p < 0.05 by Tukey’s multiple comparison test. (G and H) Mean speeds of intracellular GLUT4 movements before and after insulin stimulation (100 nM) in the presence or absence of LY294002 pretreatment in either 3T3L1 adipocytes expressing myc-GLUT4-mCherry (G) or the reconstitution models (H) under the indicated conditions (*n* = 4 cells). Data with error bars are the mean ± SEM. ∗p < 0.05 by Mann-Whitney *U* test. See also Figure S4.

**Figure 3:**
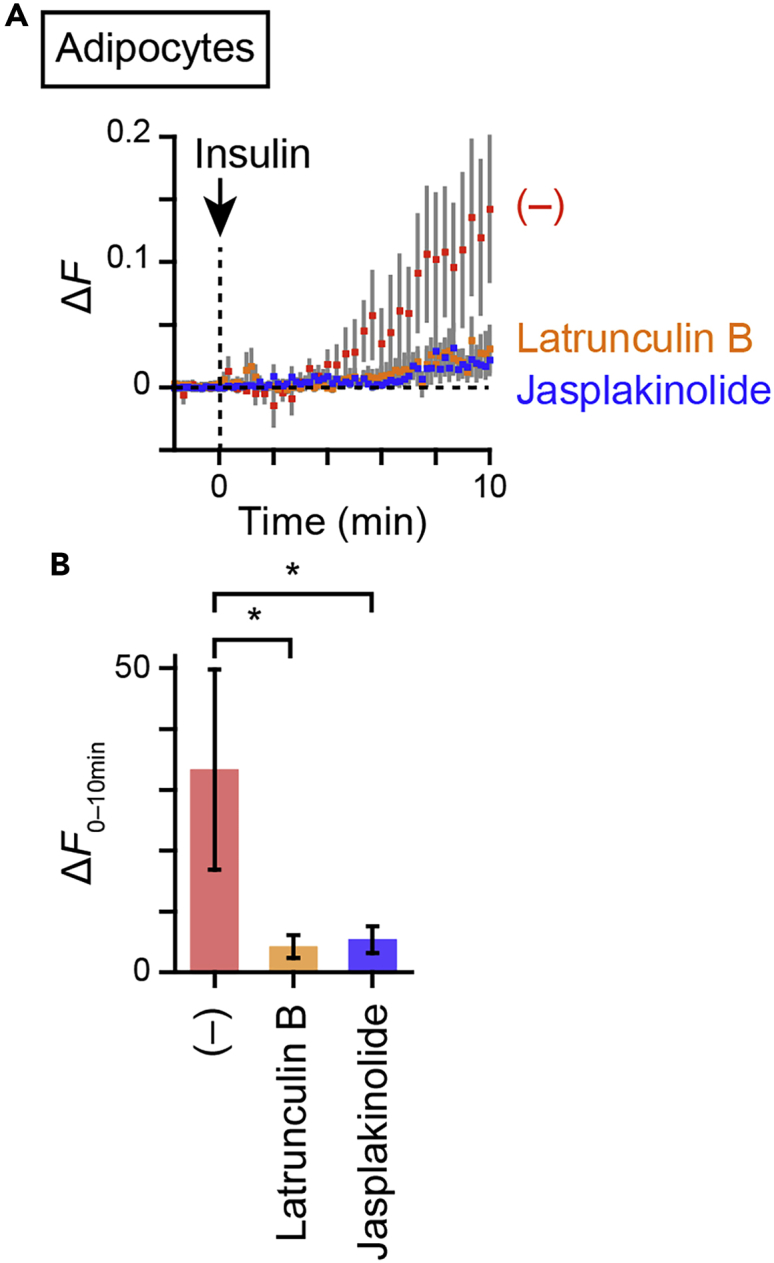
Role of actin dynamics in heterotypic endosome fusion in 3T3L1 adipocytes Changes in Δ*F* (A) and Δ*F*_0–10min_ (B) in 3T3L1 adipocytes expressing myc-GLUT4-mCherry and transferrin receptors. Cells that had been labeled with BODIPY/streptavidin-conjugated anti-myc antibodies were first treated with 5 μg/mL of transferrin-biotin for 5 min, washed for 5 min, and then treated without (red) or with 20 μM of latrunculin B (orange) or 5 μM of jasplakinolide (blue) for 5 min, followed by stimulation with insulin (100 nM) at time 0. Data with error bars are the mean ± SEM. ∗p < 0.05 by *Mann-Whitney U test* (*n* = 4–6 cells).

With this ratiometric approach allowing more precise quantification of heterotypic fusion, we analyzed regulations of the processes within cells. First, we observed this insulin-responsive heterotypic endosomal fusion in fully differentiated 3T3L1 adipocytes as in skeletal myofibers, a response completely inhibited by the PI 3-kinase inhibitor LY294002 (Figures 2A–2C). We previously reconstituted insulin-responsive GLUT4 behavior with respect to GLUT4 movement, which consists of insulin-responsive liberation of static GLUT4, in undifferentiated 3T3L1 fibroblasts by exogenously expressing three proteins that were markedly upregulated upon differentiation, i.e., GLUT4, sortilin, and AS160 (hereafter, referred to as “reconstitution models”) (Hatakeyama and Kanzaki, 2011, 2013a; b; Hatakeyama et al., 2019). Herein, we found that insulin can elicit heterotypic fusion in the reconstitution models, which was also completely inhibited by LY294002 (Figures 2D–2F). Consistent with our previous observations, QD-based single-molecule tracking of GLUT4 movement demonstrated insulin-responsive liberation of static GLUT4 in 3T3L1 adipocytes (Figure 2G) and the reconstitution models (Figure 2H) to be completely inhibited by LY294002. Note that these data fully parallel insulin-responsive heterotypic endosomal fusion. In addition, AS160 is also required for endosomal fusion, because the adipocytes depleted of AS160 by electroporation with AS160 siRNA (Figure S4A) failed to show insulin-responsive heterotypic fusion, and rescue of AS160 expression in adipocytes by electroporation with both mouse-specific AS160 siRNA and HaloTag-AS160, the sequences of which are based on human AS160, restored insulin-responsive heterotypic fusion (Figures S4B and S4C). Again, the insulin-responsive heterotypic fusion fully paralleled the insulin-responsive liberation of static GLUT4 in these cells, i.e., insulin markedly induced liberation of GLUT4, which failed in AS160-depleted cells (Figure S4D). We also confirmed AS160 to be required for both heterotypic fusion and GLUT4 liberation in the reconstitution models (Figures S4E–S4G), i.e., obvious insulin-responsive heterotypic fusion and, consistently with a previous study, GLUT4 liberation were observed only in cells expressing both sortilin and AS160. As demonstrated previously, sortilin is a critical factor for generating static GLUT4 behavior but additional AS160 is necessary for liberation of the static GLUT4 in response to insulin (Figure S4G) (Hatakeyama and Kanzaki, 2011). Heterotypic fusion in fibroblasts expressing sortilin but not AS160 again occurred in parallel, i.e., insulin did not induce heterotypic fusion in the cells (Figures S4E and S4F). These overall observations indicate that insulin stimulates heterotypic fusion between GLUT4–vesicles and TfR-containing endosomes in 3T3L1 cells, as it does in mouse skeletal myofibers (Hatakeyama and Kanzaki, 2017). These whole-cell analyses also revealed that significant heterotypic fusion between GLUT4-vesicles and TfR-containing endosomes occurs promptly, especially around the perinuclear region, in 3T3L1 cells (Figures 1 and 2), where the GLUT4 storage compartments are mainly located (Fujita et al., 2010). Interestingly, the actin polymerization inhibitor, latrunculin B, and the actin polymerization inducer, jasplakinolide, both of which inhibit actin dynamics and insulin-responsive GLUT4 translocation in 3T3L1 adipocytes (Kanzaki and Pessin, 2001), inhibited insulin-responsive increases in Δ*F* (Figure 3), indicating actin dynamics to be a critical factor in the acute heterotypic fusion occurring in response to insulin stimulation.

### Technique 2: Relationship between heterotypic fusion and GLUT4 liberation

**Figure 4:**
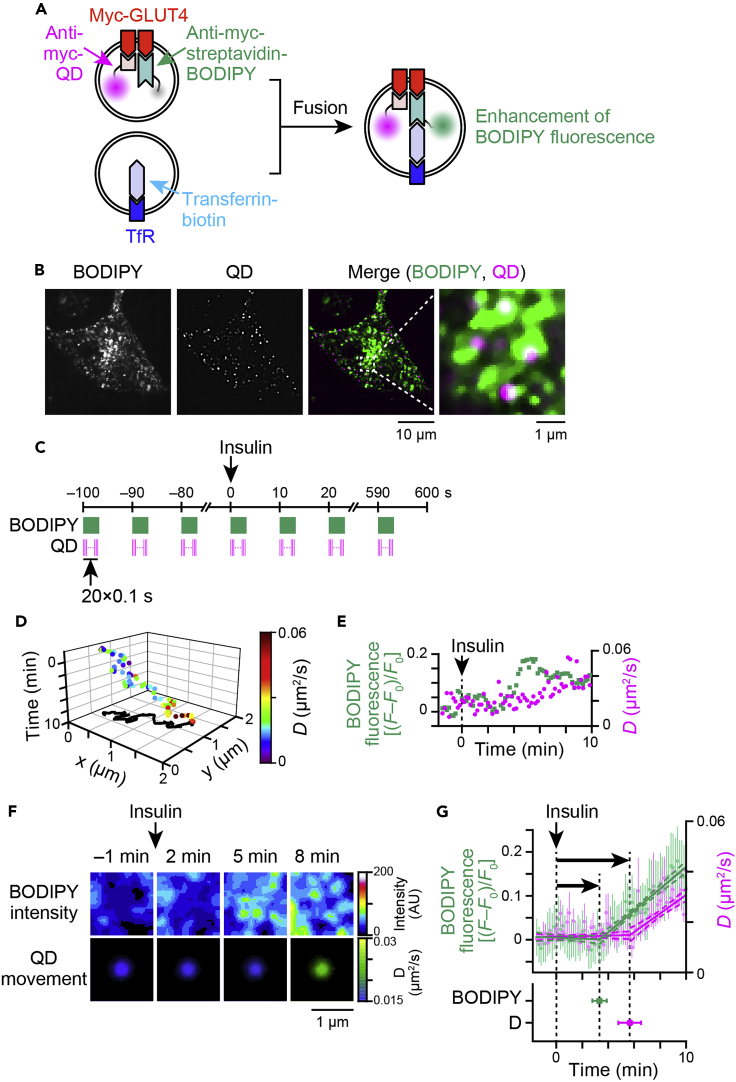
Relationship between heterotypic fusion and GLUT4 liberation (A) Schema for simultaneous detection of heterotypic fusion and GLUT4 movement. Before stimulation, myc-GLUT4 was labeled with BODIPY/streptavidin-conjugated anti-myc antibodies and QD-conjugated anti-myc antibodies. Separately, TfR was labeled with transferrin-biotin. Under these conditions, the green fluorescence of BODIPY but not the red fluorescence of QD in myc-GLUT4-containing vesicles is quenched because of the aromatic residues of streptavidin. After myc-GLUT4-containing vesicles fuse with TfR-containing vesicles, biotin-streptavidin binding dequenches BODIPY fluorescence making increases in the green, but not in the red, fluorescence detectable. QD-based single molecule imaging was simultaneously performed by tracking QD-labeled myc-GLUT4 molecules. Given the wide absorption spectrum and large Stokes shift of QD, simultaneous visualization of both fluorescent molecules was achieved. (B) Snapshots of BODIPY (*left*) and QD (*middle*) fluorescence and the merged image (*right*) in 3T3L1 fibroblasts expressing myc-GLUT4-mCherry, HA-sortilin and HaloTag-AS160. (C) Imaging protocol for simultaneously analyzing heterotypic fusion (by BODIPY fluorescence) and GLUT4 movement (by QD fluorescence). Both fluorescences were acquired every 10 s, and each image was acquired with 1 × 2 s exposure for BODIPY and 20 × 0.1 s exposure for QD. Diffusion coefficients every 10 s were calculated with QD fluorescence during 20 frames. (D) Example of a QD movement. This graph represents the mean position of QD (*x axis* and *y axis*), time (*zaxis*), and the diffusion coefficient at each time point (color; displayed right). Projection of the movement onto the *xy*-plane is also shown. (E) Changes in diffusion coefficients (*magenta circles*) and BODIPY fluorescence along the QD trajectory (*green squares*) calculated by using one QD movement shown in (D). (F) Images representing mean changes in BODIPY fluorescence (*upper* in F) and diffusion coefficients (*lower* in F) in response to insulin stimulation (100 nM) at time 0 (*n* = 60 molecules). Pseudocolor codings, displayed on the right, are used to represent the BODIPY intensity and the diffusion coefficient of QD, respectively. (G) Mean changes in the diffusion coefficient (*magenta circles*) and BODIPY fluorescence along the QD trajectory (*green squares*) (*upper*). Error bars represent SEM. Solid and dashed lines represent fitting curves with piecewise functions and their 95% confidence limits, respectively. Ninety-five percent confidence intervals for the intersections of piecewise functions for BODIPY fluorescence and diffusion coefficients are also shown (*lower*).

Next, to assess the relationship between heterotypic fusion and GLUT4 liberation in response to insulin, we established a simultaneous imaging system for monitoring both heterotypic fusion and individual GLUT4 movements by combining our fusion sensor with GLUT4 nanometry employing QD (Fujita et al., 2010) (Figure 4A). The broad absorption spectrum and the large Stokes shift of QD allowed us to fully visualize these two fluorescent molecules simultaneously (Figure 4B). To minimize photobleaching of BODIPY because of the laser intensity required for single molecule imaging of QD, we acquired time series images with intervals of 10 s, and at each time point, we acquired 1 frame of BODIPY fluorescence and 20 frames of QD fluorescence at 10 frames/s (Figure 4C). We tracked QD fluorescence during acquisition of the 20 frames at each time point and calculated the diffusion coefficient transition during this period (Figure 4D). Then, we measured BODIPY fluorescence around the mean QD fluorescence position at the corresponding time points (Figure 4E). With this approach, we were able to demonstrate obvious increases in both BODIPY fluorescence and the diffusion coefficient after several minutes of insulin stimulation by averaging these values for numerous QD signals (Figures 4F and 4G). We fitted the curves by applying piecewise functions and found that the onset of increases was much more rapid for BODIPY fluorescence than for the diffusion coefficient (Figure 4G). This indicates that heterotypic endomembrane fusion of GLUT4-vesicles with TfR-containing endosomes precedes GLUT4 liberation from its static status in response to insulin.

Given that TfR molecules are being briskly recycled among the plasma membrane, early/sorting endosomes, and endocytic recycling compartment (Mayle et al., 2012), our data at this stage do not allow us to specifically identify the TfR-positive endosomes undergoing acute fusion with GLUT4-vesicles upon insulin stimulation. Nevertheless, considering the absolute necessity of PI 3-kinase and AS160 for both heterotypic fusion of GLUT4-vesicles and GLUT4 liberation, these two events clearly arose from the AS160-associating GLUT4-vesicles (Larance et al., 2005), a process regulated by well-established proximal intracellular signals elicited by insulin (Kanzaki, 2006). The simultaneous imaging data presented herein strongly suggest that GLUT4 molecules within static GLUT4-vesicles are mobilized upon mixing with the TfR-containing endosomes immediately after heterotypic endosomal fusion in response to insulin.

### Technique 3: Panoramic landscape of intracellular trafficking activities of GLUT4 and TfR

**Figure 5:**
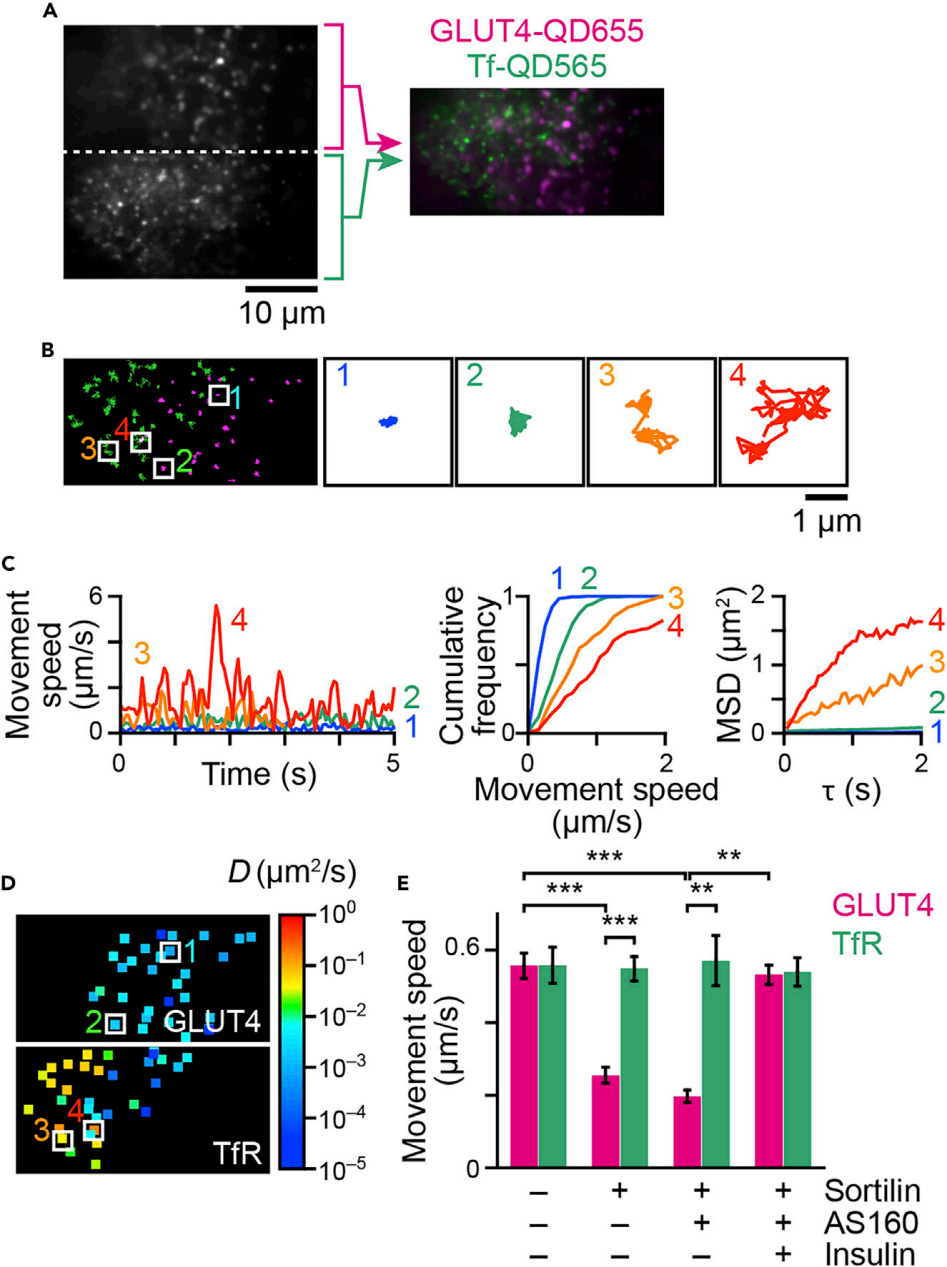
Simultaneous detection of intracellular movement of GLUT4 and transferrin receptor (A) Snapshot of QD655-labeled myc-GLUT4 and QD565-labeled transferrin in a 3T3L1 fibroblast expressing myc-GLUT4-ECFP and HA-sortilin projected onto a single camera. Pseudocolored images of the upper and lower halves (aligned and merged) are also shown (*right*). (B) Movements of QD655-labeled myc-GLUT4 (*magenta*) and QD565-labeled transferrin (*green*) in a cell shown in (A). Single particle tracking was performed separately, and the trajectories obtained were merged. Magnifications of four particles indicated by boxes are also shown. (C) Time-courses of the movement speed (*left*), distributions of the speed (*middle*), and MSD curves (*right*) of the four particles shown in (B). (D) Diffusion coefficient maps in the cell shown in (A). A pseudocolor coding, displayed on the right, is used to represent the diffusion coefficient of the molecule. (E) Mean movement speeds of GLUT4 (*magenta*) and TfR (*green*) in 3T3L1 fibroblasts expressing only myc-GLUT4-ECFP, myc-GLUT4-ECFP + HA-sortilin, or myc-GLUT4-ECFP + HA-sortilin + HaloTag-AS160 before and after insulin stimulation (100 nM). Data are the mean ± SEM. ∗∗p < 0.01, p < 0.001 by Tukey’s multiple comparison (*n* = 5–10 cells).

**Figure 6:**
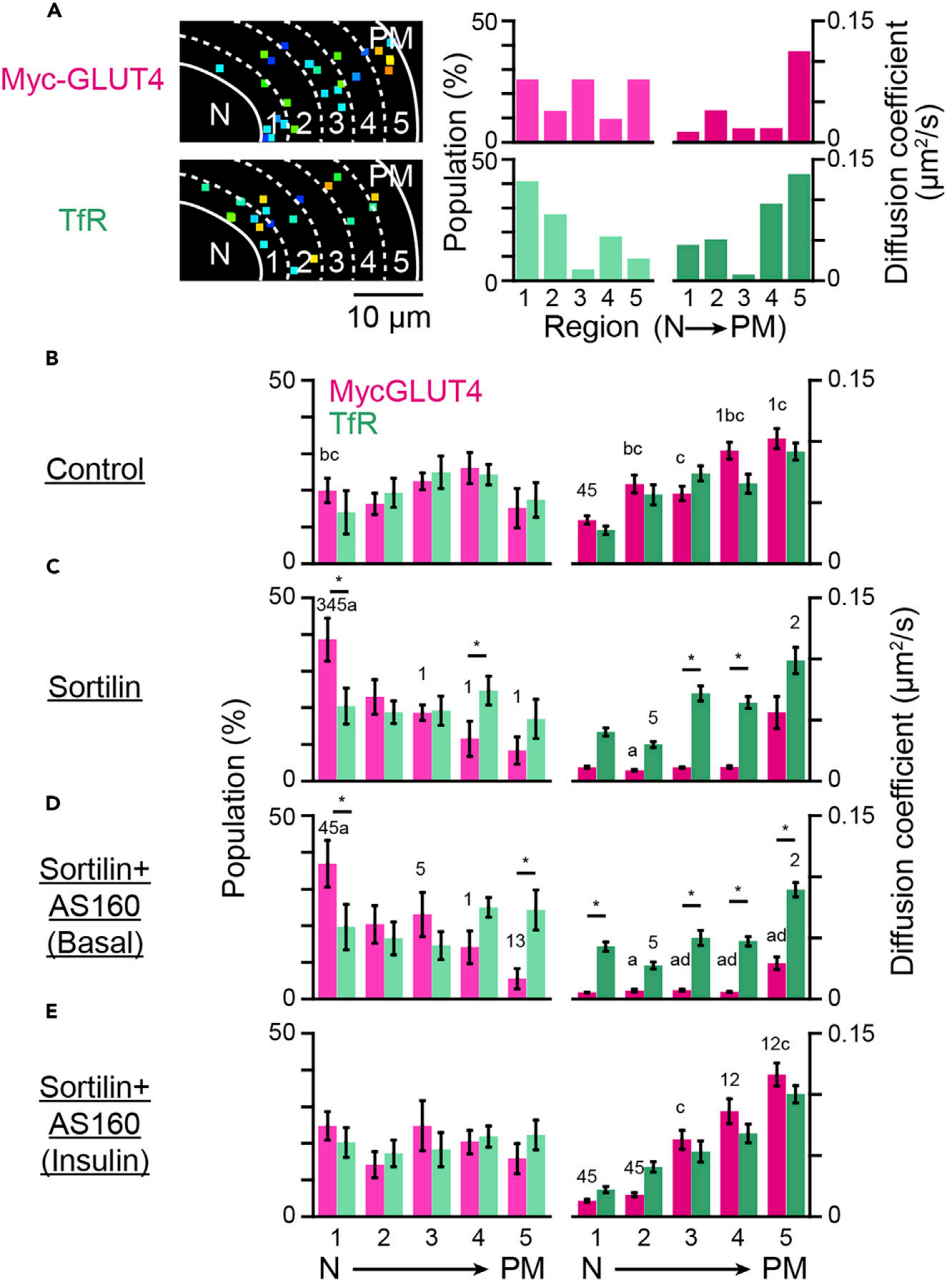
Regional analysis of movements of GLUT4 and TfR (A) Analytical scheme. We divided the cytoplasmic area into 5 equally spaced regions and calculated the population of QD-labeled molecules and the mean diffusion coefficient within each area. (B–E) Populations (*left*) and diffusion coefficients (*right*) of myc-GLUT4 (magenta) and TfR (green) in 3T3L1 fibroblasts expressing myc-GLUT4-ECFP (control, B), cells expressing myc-GLUT4-ECFP + HA-sortilin (sortilin, C), cells expressing myc-GLUT4-ECFP + HA-sortilin + HaloTag-AS160 (sortilin + AS160) before (D) and after (E) insulin stimulation (*n* = 4 cells). Asterisks, numbers and letters represent significant differences by Tukey’s multiple comparisons test. Asterisks represent significant differences between GLUT4 and TfR in the corresponding regions of the corresponding cells. Data with error bars are the mean ± SEM. Numbers represent significant differences versus the indicated region of the corresponding molecules in the corresponding cells. Letters represent significant differences between expression/treatment conditions (a–d represent versus control, sortilin, sortilin+AS160 (basal), sortilin+AS160 (insulin), respectively) of the corresponding molecules in the corresponding regions.

**Figure 7:**
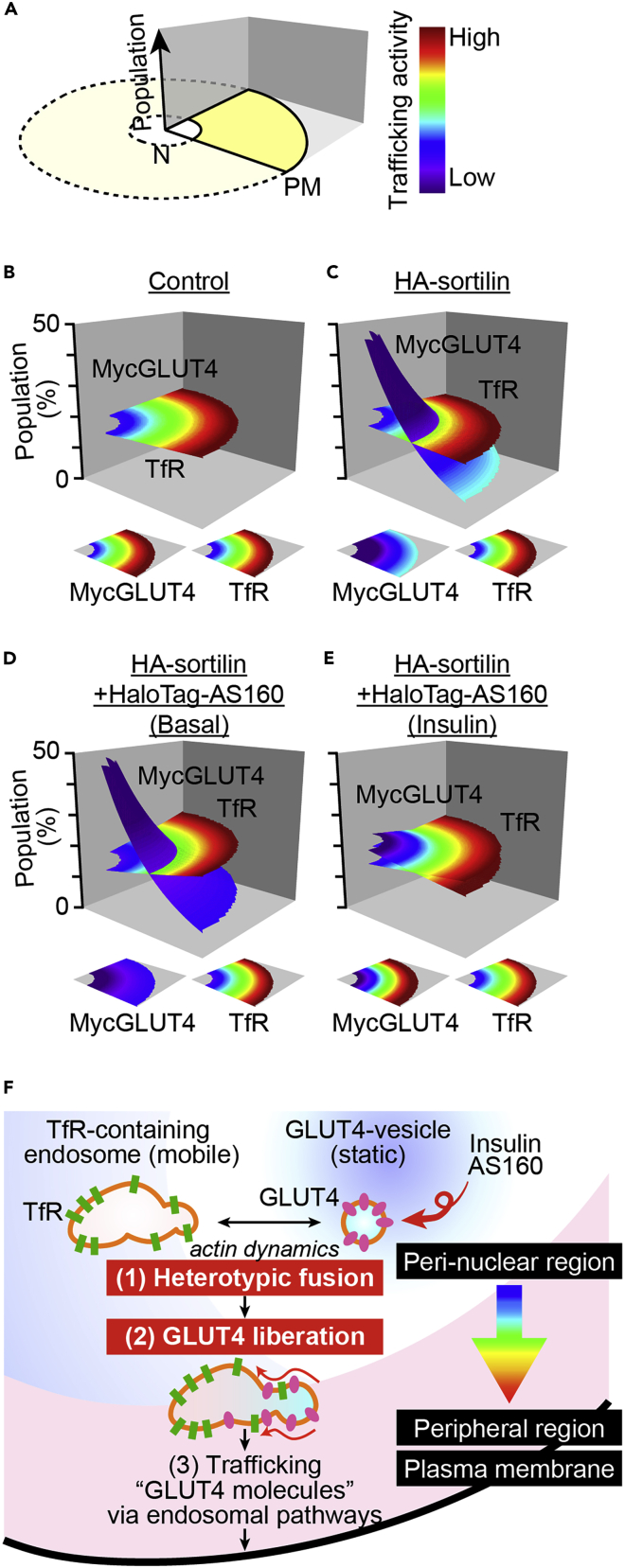
Intracellular trafficking activity maps of GLUT4 and TfR (A) Model cell and pseudocolor coding for depicting intracellular trafficking activity maps. (B–E) Intracellular trafficking activity maps constructed on the basis of the data shown in Figures 6B–6E. In each panel, the upper map represents an intersecting surface plot of trafficking activities of myc-GLUT4 and TfR. To facilitate understanding the distributions of trafficking activities, we also show contour projections of the respective molecules (*lower*). (F) Schematic depiction of the very early responses of GLUT4-vesicles in response to insulin stimulation. Insulin stimulates (1) heterotypic fusion of GLUT4-vesicles that precedes (2) GLUT4 liberation from its static status in the perinuclear region. Once GLUT4 molecules enter the TfR-containing endosomes in great excess, they appear to translocate to the plasma membrane by taking advantage of the brisk trafficking properties possessed by TfR-positive endosomes.

Given that a significant fraction of GLUT4-vesicles promptly fuses with TfR-containing endosomes, especially around the perinuclear region (Figures 1 and 2), our observations allow us to hypothesize that, once GLUT4 molecules enter the endosomes from static GLUT4-vesicles, thereby exporting GLUT4 molecules rather than delivering GLUT4-vesicles, the overall endosomal trafficking itinerary including the endocytic recycling compartment system contributes to effectively translocating GLUT4 from its intracellular storage compartments *en route* to the cell surface. If so, the trafficking properties of GLUT4 molecules in response to insulin would presumably be immediately governed by rules pertaining to the recipient endosomes containing TfR that exhibits high velocity with higher diffusion coefficients of the membrane cargo proteins (Fujita et al., 2010; Hatakeyama and Kanzaki, 2011). To directly address this question, we utilized dual-color simultaneous single-molecule tracking of TfR and GLUT4 labeled with two QDs, each having a distinct color (Hatakeyama and Kanzaki, 2013a). This approach allows us to compare intracellular movements of TfR and GLUT4 within the same cell simultaneously and directly (Figures 5A–5D). By employing this approach in cell-based reconstitution models, we first confirmed our previous observations with single-color tracking of either TfR or GLUT4 within a cell, i.e., selective restriction of GLUT4 movement in the presence of sortilin and insulin-responsive liberation of GLUT4 in the presence of additional AS160 without significant changes in TfR movement (Figure 5E). To quantify the regional dependence of movement properties of TfR and GLUT4 within a cell in further detail, we next extracted subcellular regional information regarding the numbers and behaviors of molecules (defined as “trafficking activities”) by dividing the intracellular area into 5 equally spaced regions between the nuclear envelope (nucleocytoplasmic boundary) and the plasma membrane (Figure 6A). We calculated the numbers of these molecules and mean diffusion coefficients within each area (i.e., regional trafficking activities, Figure 6A). Based on these data, we can obtain quantitative information on intracellular regional trafficking activities by depicting these data as individual graphs, separately displaying populations (Figures 6B–6E, *left*) and their diffusion coefficients (Figures 6B–6E, *right*). In control 3T3L1 fibroblasts expressing only myc-GLUT4, both TfR and GLUT4 showed near-even dispersions within an entire area and a higher diffusion coefficient toward the cell periphery than in the perinuclear region (Figure 6B). However, sortilin expression resulted in a marked increase in the perinuclear segregation of GLUT4 that exhibits a very low diffusion coefficient with no obvious changes in those of TfR (Figure 6C). Remarkable discrimination between TfR and GLUT4 was also apparent in cells expressing both sortilin and AS160 under basal conditions (without insulin stimulation, Figure 6D). In contrast, insulin stimulation made behavioral characteristics of TfR and GLUT4 indistinguishable, i.e., insulin stimulation shifted GLUT4 distributions to near-even dispersions within an entire area and increased the diffusion coefficient, especially in the peripheral regions (Figure 6E), implying that the GLUT4 molecules had joined the TfR itinerary immediately after insulin stimulation. These graphs allow us to obtain quantitative information regarding regional populations and their diffusion coefficients, but they are quite complicated and difficult to interpret. Therefore, to comprehensively evaluate intracellular regional trafficking activities, we reconstructed panoramic landscapes of trafficking activities (Figure 7). We referred to these as “trafficking activity maps,” featuring pseudocolor coding in a simple concentric regional map of the cell outlines derived from a circle divided into quarters (Figure 7A). The resulting maps represent multiple key aspects of trafficking activities, i.e., subcellular localization in terms of concentric zones (*x axis* and *y axis*), populations (*z axis*) and the diffusion coefficients (*color*) of TfR and GLUT4 (Figures 7B–7E). Intriguing observations from the trafficking activity maps are discussed below.

## Discussion

Our previous and current live-cell imaging analyses of GLUT4 behavior demonstrated the crucial initial events that allow full initiation of the GLUT4 trafficking itineraries, i.e., liberation of static GLUT4 and heterotypic fusion. Derangements of the unique GLUT4 trafficking system are related to insulin resistance, and comprehensive evaluation of the initial events is critical for obtaining a deeper understanding of physiology and pathophysiology relating to GLUT4 trafficking. Herein, in addition to demonstrating the occurrence of heterotypic fusion in 3T3L1 cells, we precisely and comprehensively quantified and also displayed the regulation, interrelationships, and behaviors of the crucial initial components of insulin-responsive GLUT4 trafficking in these cells. We applied highly rigorous quantitative approaches that combine three types of analyses based on dual-color live-cell imaging approaches and demonstrated crucial mechanisms regulating the initial events of GLUT4 behavior, none of which had been revealed by the approaches used in previous studies. As to the unexpected realization of the importance of heterotypic fusion, we originally identified this phenomenon in skeletal myofibers, albeit with analyses limited to subcellular regions, which show increases in the size of small GLUT4-containing structures in response to insulin, as demonstrated using STED microscopy (Hatakeyama and Kanzaki, 2017). Despite GLUT4 translocation processes having long been extensively analyzed, especially in 3T3L1 adipocytes (Kanzaki, 2006; Klip et al., 2019), such early heterotypic endomembrane fusion triggering GLUT4 redistribution in response to insulin had not previously been demonstrated, possibly because of the lack of suitable analytical methods and the dense perinuclear localization of GLUT4, which hinders visualization of individual small GLUT4-containing structures in living cells. In addition, the events occurring deep inside cells depend on extremely complicated processes involving highly heterogeneous structures composed of a wide variety of membrane lipids and proteins, also making detailed measurements difficult. Our approaches allowed us to overcome these drawbacks and provide meaningful insights into the initiation of insulin-responsive GLUT4 translocation, as discussed in the following paragraph, with an entirely unprecedented view of intracellular trafficking properties that other available techniques cannot directly address. Being able to analyze heterotypic fusion within all regions of the cell, as in the present study with ratiometric analysis, contributed to demonstrations of prompt insulin-responsive heterotypic fusion predominantly around the perinuclear region, where the GLUT4 storage compartments are mainly located. In the previous study that relied solely on BODIPY fluorescence, we could not fully rule out the possibility that enhancement of BODIPY fluorescence was because of incorporation of endocytosed GLUT4 after exocytosis into the TfR-containing endosomes. However, we can now exclude this possibility with confidence based on analyzing subcellular regional dependence, because enhancement of BODIPY fluorescence in response to insulin occurs predominantly in the regions near the plasma membrane. Rather, heterotypic fusion serves as one of the initial triggers for insulin-responsive GLUT4 translocation, causing direct fusion between GLUT4 vesicles from the storage compartments and TfR-containing endosomes.

Combining our previous (Fujita et al., 2010; Hatakeyama and Kanzaki, 2011, 2013a) and present results clearly illustrates the very earliest events regulating insulin-responsive GLUT4 translocation (Figure 7F). First, statically-retained GLUT4 molecules within storage compartments promptly undergo heterotypic fusion with TfR-containing endosomes in response to insulin, and these fusion processes are dependent on PI3K, AS160, and actin dynamics (Figures 1, 2, 3, and 4). Then, the trafficking activity of the GLUT4 molecules instantly increases to levels similar to that of TfR (Figures 5, 6, and 7). This indicates that GLUT4 molecules are promptly incorporated into and exploit the TfR trafficking itinerary at least under conditions of continuous insulin presence. Thus, we obtained compelling evidence that insulin-induced heterotypic endosomal fusion originating from GLUT4-vesicles is found in both adipocytes and myofibers (Hatakeyama and Kanzaki, 2017) and that both cell types possess a highly efficient insulin-responsive GLUT4 translocation system which operates via heterotypic fusion. More importantly, the present imaging analyses shed light on the physiological significance of insulin-induced GLUT4 ‘protein trafficking,’ mediated through a general anterograde endosomal pathway in both adipocytes and myofibers. Thus, our present findings challenge the canonical concept derived from “GLUT4-vesicle trafficking;” i.e., most GLUT4-vesicles are destined to directly reach their final destination, the plasma membrane. Rather, insulin-induced GLUT4 translocation is also achieved at least in part via endosomal “GLUT4-molecule” trafficking from the perinuclear static GLUT4 storage compartment via fusion with a subset of traveling endosomes, which can contain TfR, upon insulin stimulation (Figure 7F).

In regulating heterotypic fusion, as expected, PI 3-kinase activity and AS160, a member of Tbc1d family of Rab GTPase-activating proteins (GAPs) (Sano et al., 2003), are critical factors, as shown in both differentiated 3T3L1 adipocytes and reconstitution model cells (Figure 2 and S4). AS160 exhibits potent GAP activity affecting multiple Rab proteins, including Rab8, 10, 14, and 28 (Miinea et al., 2005; Zhou et al., 2017), all of which are involved in endomembrane trafficking (Chen and Lippincott-Schwartz, 2013; Langemeyer et al., 2018; Sano et al., 2007; Ungermann and Kummel, 2019). Thus, once AS160 is activated, upon catalytic release from Rab GAP-mediated suppression, several downstream effectors cooperatively participate, not only in the canonical fusion event at the plasma membrane (Chen et al., 2012) but also, as revealed herein, in the initial heterotypic endosomal fusion processes. Indeed, insulin-dependent GLUT4 translocation requires multiple Rab proteins, e.g., Rab8, 10, and 14, that may function at divergent steps of the GLUT4 trafficking processes (Brewer et al., 2016a, 2016b; Chen et al., 2012). Although the molecular mechanisms underlying heterotypic fusion are presently unknown, various fusion and tethering machineries, capable of being regulated by RabGTPases, might be intimately involved. These include SNARE proteins, e.g., VAMP3 (Riggs et al., 2012), VAMP4 (Sadler et al., 2015), syntaxin 6, syntaxin 16, syntaxin 13, Vti1a (Brandhorst et al., 2006), and VAMP8 (Antonin et al., 2000; Behrendorff et al., 2011), which also reportedly function in endosomal fusion processes and GLUT4 translocation (Randhawa et al., 2004; Williams and Pessin, 2008; Zhao et al., 2009). In fact, v-SNAREs involved in insulin-responsive GLUT4 translocation appear to be complex because insulin-induced GLUT4 translocation can reportedly be inhibited only by disruption of multiple v-SNAREs, not by disrupting VAMP2, VAMP3, or VAMP8 individually (Zhao et al., 2009). This evidence underscores the apparent importance of the relatively promiscuous fusion behavior of GLUT4-vesicles upon insulin stimulation toward heterotypic endosomes, clearly providing multiple alternative trafficking routes to the plasma membrane, the final destination. Moreover, given the emerging importance of multi-subunit tethering complexes in various endosomal fusion events, recently identified tethering complexes, such as CORVET (van der Beek et al., 2019), may also be involved in heterotypic endomembrane fusion events triggered by GLUT4-vesicles upon insulin stimulation. In addition, TUG, a GLUT4- tethering protein involved in insulin-dependent GLUT4 release from its storage compartments, may participate in the very early phases of GLUT4-vesicle regulation, though insulin-dependent regulation of TUG appears to be mediated through the Rho-family small GTPase TC10, but not the PI 3-kinase, pathway (Bogan et al., 2012; Habtemichael et al., 2018). Importantly, we found that both latrunculin B and jasplakinolide inhibit insulin-responsive endosomal fusion (Figure 3), suggesting an essential role of actin dynamics rather than the cytoskeletal F-actin structures in this insulin-responsive endomembranous event. Given that the heterotypic endosomal fusion precedes GLUT4 liberation from its static status in response to insulin (Figure 4), proper regulation of actin dynamics at the perinuclear region is crucial for the initial insulin action on GLUT4 redistribution. Consistently, we previously reported that insulin induces dynamic actin remodeling in both the perinuclear region (Kanzaki et al., 2001) and just beneath the plasma membrane (cortical regions) (Kanzaki and Pessin, 2001). In addition, such actin dynamics play important roles in insulin-responsive GLUT4 translocation (Kanzaki, 2006). Thus, although the precise molecular mechanisms underlying the insulin-responsive heterotypic endosomal fusion of GLUT4-vesicles, relying on perinuclear actin dynamics, remain unclear, our imaging analysis extends previous indirect evidence suggesting the importance of actin dynamics in the perinuclear region by showing that insulin-responsive fusion of GLUT4-vesicles requires these dynamics. The balance between polymerization and depolymerization of actin and actions in concert with those of the other aforementioned machineries appear to be critical for the initial event of insulin-induced GLUT4 translocation processes. Further analyses are essential for understanding the importance of endosomal fusion events in response to insulin and their underlying mechanisms.

What is the fate of GLUT4 and TfR after heterotypic fusion? Previous studies demonstrated that TfR does not redistribute to the plasma membrane to the same extent as GLUT4 after insulin stimulation (El-Jack et al., 1999) and most vesicles containing IRAP, one of the GLUT4-vesicle-resident proteins, arriving at the plasma membrane in response to insulin have no TfR (Chen et al., 2012). However, the latter report also demonstrated modest amounts (>30%) of IRAP-containing vesicles approaching the plasma membrane to have TfR (Chen et al., 2012), strongly suggesting that a significant amount of GLUT4 can co-translocate with TfR to the plasma membrane after insulin stimulation. Moreover, it should be noted that GLUT4-containing vesicles are incorporated into TfR-containing endosomes only upon heterotypic fusion and that such GLUT4 loading will not affect either the frequency or the number of TfR-containing endosomes approaching the plasma membrane. In this regard, some of the TfR-containing endosomes may function as transit hubs between GLUT4 storage compartments and the plasma membrane, and GLUT4 molecules might translocate to the plasma membrane via multiple rounds of fusion and fission with these transit hubs. In this case, GLUT4 and TfR are not necessary for co-translocation to the plasma membrane.

Constructing “trafficking activity maps” leads to another intriguing observation that intracellular trafficking activities are apparently governed by subcellular areas, with activities tending to be higher toward the cell periphery than in the perinuclear region, even for TfR (Figures 6 and 7). GLUT4 exhibits such a subcellular trafficking activity gradient particularly in cells expressing sortilin and AS160, which is in excellent agreement with our previous immunostaining data showing their perinuclear region accumulations (Hatakeyama and Kanzaki, 2013b). However, it is surprising that such region-dependent trafficking activity is also observed for TfR. We cannot exclude the possibility of the perinuclear trafficking activity caused by cellular geometry being underestimated, as perinuclear regions are slightly thicker than outer peripheral regions, although such an underestimation would be negligible. Rather, it is highly likely that the cellular architecture comprising organelle positions and their distributions (i.e., perinuclear Golgi apparatus segregation) (Rafelski and Marshall, 2008), as well as subcellular microrheological properties (Tseng et al., 2002) are involved in the geometric gradients of intracellular trafficking activity quantitatively detailed herein. Future studies need to focus on elucidating further details of this important issue.

Trafficking activity maps also allow us to intuitively understand subtle alterations of GLUT4 trafficking activities in the basal state in cells co-expressing sortilin and AS160 as compared to those in cells expressing solely sortilin (Figures 7C and 7D). This observation was already made using single-color GLUT4 nanometry in our previous (Hatakeyama and Kanzaki, 2011) and present (Figure S4) studies, i.e., very subtle reductions in GLUT4 behavior in undifferentiated fibroblasts expressing sortilin and AS160 compared to those expressing solely sortilin and subtle increases in GLUT4 behavior in differentiated adipocytes depleted of AS160 in the basal states; however, the difference in GLUT4 trafficking activity in the peripheral regions of the sortilin alone group (peripheral sky blue in Figure 7C) versus the sortilin plus AS160 group (peripheral dark blue in Figure 7D) is visually and readily understandable. These observations suggest the involvement of AS160 in the basal GLUT4 retention property, which is consistent with the results of previous biochemical studies (Brewer et al., 2011; Larance et al., 2005; Sano et al., 2007). Regarding heterotypic endomembrane fusion, it might be that the fusion activity in AS160-depleted cells in the basal state is higher than that in control cells, but direct comparison of the fusion processes between these two cell types is quite difficult because of the technical limitations of our current sensor molecule. We anticipate that future analyses with improved sensors will clarify this point.

In any case, once insulin induces the release of GLUT4 molecules from their static states in perinuclear regions via fusion with the subset of TfR-containing endosomes, GLUT4 molecules become behaviorally indistinguishable from TfR. These results imply that the GLUT4 molecules converge with the TfR trafficking route immediately after insulin stimulation, and then behave equivalently, at least under continuous insulin conditions, despite sortilin and AS160, GLUT4 trafficking regulators, being adequately expressed. In addition, in 3T3L1 fibroblasts, both subcellular distributions and diffusion coefficients of GLUT4 and TfR were essentially identical, irrespective of insulin stimulation. Taking these results together, despite the existence of several sorting motifs in GLUT4 itself, including C-terminus acidic dileucine followed by the TELEY sequence (Bernhardt et al., 2009; Cope et al., 2000; Shewan et al., 2000), GLUT4 itself appears to have an intrinsic capability for behaving similarly to TfR, thereby circulating in essentially the same trafficking pathways under certain conditions. Nevertheless, upon insulin withdrawal in adipocytes, released GLUT4 molecules, but not TfR, are efficiently and distinctly retrieved into the intracellular retrograde trafficking pathway, passing through early and recycling endosomes en route to a specialized part of TGN-possessing golgin-97 (Hatakeyama and Kanzaki, 2011). The retrieved GLUT4 molecules are then repackaged into insulin-responsive GLUT4-vesicles (Pan et al., 2017; Proctor et al., 2006; Shewan et al., 2003), exhibiting a stationary property, that are notably located in perinuclear regions where they await the next round of insulin stimulation (Fujita et al., 2010).

Derangement of intracellular GLUT4 behavior and defects in insulin signaling are suggested to be etiologically related to insulin resistance (Fujita et al., 2010; Hoehn et al., 2008). Notably, our present approaches can readily be applied to other trafficking molecules, and we anticipate that combining our multiple discrete live-imaging techniques will contribute to a more comprehensive understanding of the spatiotemporal regulation of cellular logistics and related dysfunctions.

### Limitations of the study

Of course, our present approaches have limitations and there is room for improvements. First, being based on the usage of biotin-streptavidin binding, our current fusion sensor cannot analyze post-fusion events continuously because the complex formed between myc-GLUT4 and TfR cannot be dissociated. Although overcoming this drawback is quite difficult because such measurements require sensor molecules that dissociate reversibly with high rate constants in a process that can be measured with high sensitivity, highly sensitive ways to detect molecular interactions such as NanoBRET (Dale et al., 2019), based on a precise molecular design, may provide a solution to this challenge. Second, although actual intracellular movement takes place in three-dimensional space, we regard it as two-dimensional movement for technical simplicity, which may underestimate actual movement. We believe that this underestimation is negligible because the present QD-based analyses were of relatively brief duration (0.5 s at most), and if a certain molecule moves vertically across the optical section (∼1 μm) during this time period, its velocity would be >2 μm/s, a very rare situation (see Figures 2G and 2H). However, it may be desirable to measure behaviors with three-dimensional tracking in some instances (Fujita et al., 2010; Gardini et al., 2018; Hatakeyama and Kanzaki, 2011, 2013a, 2013b, 2017; Hatakeyama et al., 2019; Hatakeyama et al., 2017). Finally, operations for constructing activity maps consist of multiple time-consuming steps including single molecule imaging, tracking, and evaluation. Implementing an automated system (Hiroshima et al., 2020) would improve the efficiency of these processes.

## STAR★Methods

### Key resources table

**Table:** 

REAGENT or RESOURCE	SOURCE	IDENTIFIER
**Antibodies**		

Rabbit Anti-AS160 polyclonal antibody	Abcam	Cat#62487, RRID: AB_956350

**Chemicals, peptides, and recombinant proteins**		

Latrunculin B	Calbiochem	Cat#428020
Jasplakinolide	Calbiochem	Cat#420107
Streptavidin	ProSpec	Cat#PRO-791
BODIPY FL-NHS	Thermo	Cat#D2184
2-Mercaptoethylamine	Thermo	Cat#20408
Sulfo-SMCC	Thermo	Cat#22622
LY294002	Calbiochem	Cat#440202
Lipofectamine 3000 transfection reagent	Thermo	Cat#L3000008
Cellmatrix Type IV collagen	Nitta gelatin	Cat#638-05921
Transferrin-biotin	Thermo	Cat#T23363
Alexa 647-conjugated transferrin	Thermo	Cat#T23366
Qdot 655 ITK Amino (PEG) Quantum dots	Thermo	Cat#Q21521MP
Qdot 565 Streptavidin conjugate	Thermo	Cat#Q10131MP

**Critical commercial assays**		

Mouse IgG1 Fab and F(ab’)2 Preparation Kit	Thermo	Cat#44980

**Experimental models: Cell lines**		

3T3-L1 cells	ATCC	Cat#CCL-92.1, RRID: CVCL_0123
MYC 1-9E10.2 hybridoma cells	ATCC	Cat#CRL-1729, RRID: CVCL_G671

**Oligonucleotides**		

AS160 siRNA #1	Tomy Digital Biology	5′-GCUCUGCGCCCGUAGACUA-3′
AS160 siRNA #2	Tomy Digital Biology	5′-GACUUAACUCAUCCAACGA-3′

**Recombinant DNA**		

HaloTag-AS160	Kazusa DNA Research Institute	Clone Name: pFN21AA0603, GenBank Accession# AB463305
Myc-GLUT4-mCherry	This paper	N/A
TfR	Fujita et al. 2010	N/A
HA-sortilin	Hatakeyama and Kanzaki 2011	N/A

**Software and algorithms**		

Fiji ImageJ	NIH	RRID:SCR_002285
ORIGIN	OriginLab	RRID:SCR_014212
SPSS	IBM	RRID:SCR_019096

### Resource availability

#### Lead contact

Further information and requests for resources and reagents should be directed to and will be fulfilled by the lead contact, Makoto Kanzaki (makoto.kanzaki.b1@tohoku.ac.jp).

#### Materials availability

This study did not generate new unique reagents.

### Experimental model and subject details

#### Cell lines

3T3-L1 fibroblasts (RRID: CVCL_0123) were grown at 37°C in an 8% CO_2_ atmosphere in DMEM containing 4.5 g/L D-glucose and 10% CS and 1% penicillin/streptomycin (100 U/mL penicillin; 100 μg/mL streptomycin). Two days after reaching confluence, the cells were differentiated into adipocytes in DMEM containing 4.5 g/L D-glucose, 10% fetal bovine serum (FBS), 500 μM isobutylmethylxanthine, 25 μM dexamethasone, 4 μg/mL insulin and 1% penicillin/streptomycin for 4 days, followed by further differentiation in DMEM containing 4.5 g/L D-glucose, 10% FBS, 4 μg/mL insulin and 1% penicillin/streptomycin for an additional 2–4 days. For transfection into fibroblasts, the cells were plated onto glass-bottom dishes (thickness 0.17 mm, Matsunami glass) and, the next day, transfected with Lipofectamine 3000 with plasmid DNAs (myc-GLUT4 fused to fluorescent proteins, HA-sortilin and HaloTag-AS160) diluted in Opti-MEM I, in accordance with the manufacturer’s instructions. For eletroporation into differentiated adipocytes, the cells were plated onto glass-bottom dishes coated with Cellmatrix Type IV collagen, and, after 24-h culture, electroporated with 10–25 μg of plasmid DNA and/or 250 pmol of AS160-targeted siRNA diluted in electroporation buffer consisting of 150 mM trehalose, 5 mM potassium phosphate buffer, 5 mM MgCl_2_, 2 mM ethylene glycol tetraacetic acid, 2 mM ATP, 25 mM HEPES-KOH (pH 7.3) and 1% DMSO with a CUY21EDITII electroporator (BEX) and an electrode (LF513-5, BEX) by applying a poration pulse at 200 V for 10 ms, followed by five pulses at −30 mV for 10 ms at 50-ms intervals. Knockdown of AS160 with the siRNA was confirmed by immunofluorescence in 3T3L1 adipocytes expressing myc-GLUT4-EGFP (Figure S4A) as previously described (Hatakeyama and Kanzaki 2011).

MYC 1-9E10.2 hybridoma cells (RRID: CVCL_G671) were maintained at 37°C in a 5% CO_2_ atmosphere in RPMI 1640 medium supplemented with 10% ultra-low IgG FBS. Anti-myc antibodies were purified from the culture supernatant with Protein G Sepharose 4 fast flow (Cytiva 17061801).

### Method details

#### Imaging of endomembrane fusion

Imaging of endosomal fusion of myc-GLUT4-containing vesicles with TfR-containing vesicles was performed using a previously described fluorometric avidin-biotin binding assay (Hatakeyama and Kanzaki, 2017), with slight modifications in 3T3-L1 cells expressing myc-GLUT4-mCherry. BODIPY/streptavidin-conjugated anti-myc antibodies were prepared with anti-myc antibodies, recombinant streptavidin, BODIPY FL-NHS, sulfo-SMCC, and 2-mercaptoethanolamine as previously described (Hatakeyama and Kanzaki, 2017). The bathing solution for all imaging experiments contained 150 mM NaCl, 5 mM KCl, 2 mM CaCl_2_, 1 mM MgCl_2_, 10 mM HEPES-NaOH (pH 7.4) and 5.5 mM D-glucose. In experiments using 3T3-L1 adipocytes, we also electroporated with TfR. Cells were treated with 4 μg/mL BODIPY/streptavidin-conjugated anti-myc antibodies for 1 h in the presence of 1 nM insulin, the concentration of which is critical for analyzing heterotypic fusion, since labeling with 100 nM insulin or without insulin results in failure to efficiently label myc-GLUT4 (data not shown). For example, the BODIPY fluorescence in the cells labeled with 100 nM insulin was surprisingly faint, as compared to that in cells labeled with 1 nM insulin, possibly due at least in part to the fluorescent property of BODIPY, i.e., high local concentrations of the fluorophore cause prominent self-quenching of the dye (Liu et al., 2019). The cells were then extensively washed and additionally incubated for at least 3 h. After incubation, the cells were treated with 5–12.5 μg/mL transferrin-biotin for 5 min and then thoroughly washed for 10 min. Some cells were treated with LY294002, latrunculin B or jasplakinolide during the washing period. Imaging experiments were performed with an inverted microscope (DMI6000, Leica) equipped with an SP8 confocal scanner, an oil-immersion objective lens (HC PL APO CS2 63×, NA 1.40), and white-light pulsed laser and hybrid detectors. Imaging was performed at ∼30°C using a stage heater and a lens heater (TOKAI HIT). Excitations were performed sequentially, at 488 nm and then at 561 nm, and the fluorescences of BODIPY and mCherry were collected at 495–565 nm and 580–650 nm, respectively. Images were acquired as a *t*-series at intervals of 10 s with Leica Application Suite X software and were processed and analyzed with Fiji ImageJ (RRID: SCR_002285). First, ratio images (BODIPY/mCherry) were obtained and cellular regions were determined in each frame. Since the hybrid detectors have very low background noise, there was no need to subtract the background. We obtained two values from the ratio images. One was the ratio value within the cellular regions (*R*), the other the area of which the ratio value was above the threshold (*S*), which was set as the top 1% ratio value within the cellular region just prior to insulin stimulation (Figure 1C). We then calculated mean *R* and *S* values of the whole cell for the frames imaged during the 2-min period before insulin stimulation (defined as *R*_0_ and *S*_0_, respectively), followed by changes in *R* (defined as Δ*R*) by *R*–*R*_0_, its normalized changes (defined as Δ*R*_normalized_) by Δ*R*/*R*_0_, and changes in *S* (defined as Δ*S*) by *S*–*S*_0_. We defined the index of insulin-responsive heterotypic fusion (Δ*F*) as the product obtained by multiplying these two values (Δ*R*_normalized_ and Δ*S*). We also represented the data with areas-under-the-curve of Δ*F* during the 10-min period after insulin stimulation (Δ*F*_0–10min_).

#### Colocalization analysis

Colocalization of once internalized myc-GLUT4 and TfR was analyzed in 3T3-L1 adipocytes expressing myc-GLUT4-mCherry and TfR. Labeling was performed as with imaging for endosomal fusion, as shown above, except for labeling of TfR with Alexa 647-conjugated transferrin instead of transferrin-biotin, and pretreatment of the BODIPY/streptavidin-conjugated anti-myc antibodies with 1 mM biotin to maximize BODIPY fluorescent intensity. After stimulation, the cells were fixed, and fluorescent images were acquired with an inverted microscope (IX81, Olympus) equipped with a laser scanner (FV1000, Olympus, RRID:SCR_016840) and an oil-immersion objective lens (PlanApo 60×, NA 1.40, Olympus). For co-localization analysis (Manders et al., 1992), background fluorescence of the acquired images was measured within the cell-free area adjacent to the cell-of-interest and its mean+3SD value was subtracted from the entire image. Then, quantitative colocalization analysis was performed with a Coloc2 plug-in (Fiji).

#### Single molecule imaging and particle tracking

QD-conjugated anti-myc antibodies were prepared with anti-myc antibodies, a Mouse IgG1 Fab and F(ab’)2 Preparation Kit, Qdot 655 ITK Amino (PEG) Quantum dots, sulfo-SMCC, and 2-mercaptoethanolamine as previously described (Hatakeyama and Kanzaki, 2017). QD labeling of myc-GLUT4 was performed as previously described (Fujita et al., 2010). Imaging experiments were performed with an inverted microscope (IX81, Olympus) equipped with an EMCCD camera (iXon ultra or iXon 887, Andor Technology), a Nipkow disk confocal unit (CSU-X1 or CSU10, Yokogawa), and an oil-immersion objective lens (UPLSAPO100×O, NA 1.4, Olympus) at ∼30°C, with both a stage heater and a lens heater. Excitation was delivered at 488 nm, and the fluorescence was collected through a 655/12 bandpass filter (Semrock) at 20 frames/s for 15 s with iQ or SOLIS software (Andor). Single-particle tracking was performed with G-Count (G-Angstrom, Sendai, Japan) in a two-dimensional Gaussian fitting mode. We tracked each particle fitted within a 13 × 13-pixel region of interest for at least 30 frames. When the signal in a frame was lost due to blinking, no fitting was performed until the bright spot reappeared; when it did not reappear within 10 frames, tracking was aborted. Mean movement speeds were calculated as previously described (Fujita et al., 2010). The speeds for individual particle movements were calculated by linear fit of the displacement during four frames. Mean speeds were first calculated for a single cell, and then averaged among cells under the same treatment conditions. We found that QD in fixed cells showed non-negligible velocities (∼0.19 μm/s), attributable to unavoidable instrumental noise. Thus, when representing mean velocities, we used corrected values instead of raw data. The corrected values were obtained by subtracting the instrumental noise.

#### Simultaneous imaging of endomembrane fusion and single molecule behavior

Myc-GLUT4 was simultaneously labeled with 4 μg/mL BODIPY/streptavidin-conjugated anti-myc antibodies and 5–10 nM QD655-conjugated anti-myc antibodies for 1 h in the presence of 1 nM insulin. Imaging experiments were performed with an inverted microscope (IX81, Olympus) equipped with an EMCCD camera (iXon ultra and iXon 887, Andor Technology), a Nipkow disk confocal unit (CSU-X1, Yokogawa), a z-drift compensator (IX81-ZDC2, Olympus), and an oil-immersion objective lens (UPLSAPO100×O, NA 1.4, Olympus) at ∼30°C. Excitations of QD655 and BODIPY were both performed at 488 nm, and their fluorescences were separated by a dichroic mirror with an edge wavelength at 594 nm and collected through 655/12 and 515/40 bandpass filters (Semrock), respectively, with iQ software. We daily acquired a bright-field snapshot of a square lattice (10 μm) test pattern in order to align the two fluorescent images. Images were acquired every 10 s with 20 frames of 50 ms exposure for QD fluorescence and 1 frame of 1 s exposure for BODIPY fluorescence. Synchronization of the laser and the cameras was achieved with an Andor Precision Control Unit. With every 20 frames of QD fluorescent images, we performed single particle tracking of the fluorescence as described above. Then, we obtained BODIPY fluorescence within 7 × 7 pixels around mean positions of the QD during 20 frames. Diffusion coefficients of individual molecules were estimated based on mean-square displacement (MSD) of the particles, which was calculated with

(Equation 1) $$MSD\left(\tau \right)=\frac{1}{N-\frac{\tau }{\Delta t}}\overset{N-\frac{\tau }{\Delta t}}{\underset{i=1}{\sum }}{\left|p_{i+\frac{\tau }{\Delta t}}-p_{i}\right|}^{2}$$

where *τ*, *N*, Δ*t* and p_*i*_ are all accessible time lags, the total number of positions measured, time interval of successive images, and position of the molecule in time frame *i*, respectively. The diffusion coefficient of the molecule was estimated by fitting the first 5 points of MSD(τ) with

(Equation 2) $$\begin{array}{l} MSD\left(\tau \right)=4D\tau +C \end{array}$$

where *D* and *C* are the diffusion coefficient and instrumental noise, respectively.

#### Dual-color simultaneous single molecule imaging of GLUT4 and TfR behaviors

Dual-color QD labeling of myc-GLUT4 and the TfR was performed with QD655-conjugated anti-myc antibodies and QD565-conjugated streptavidin as previously described (Hatakeyama and Kanzaki, 2013a). Imaging experiments were performed with an inverted microscope (IX81, Olympus) equipped with an EMCCD camera (iXon 887, Andor Technology), a Nipkow disk confocal unit (CSU10, Yokogawa), and an oil-immersion objective lens (UPLSAPO100×O, NA 1.4, Olympus) at ∼30°C. Excitations of QD565 and QD655 were both performed at 488 nm, and their fluorescences were separated by a dichroic mirror with an edge wavelength at 594 nm and collected through 560/25 and 655/12 bandpass filters (Semrock) at 20 frames/s for 15 s, respectively, with SOLIS software. We also used an emission splitting optics device to project the two fluorescences into a single EMCCD camera. We daily acquired a bright-field snapshot of a square lattice (10 μm) test pattern in order to align the two fluorescent images. Single-particle tracking was performed and the diffusion coefficient was estimated as described above except for using the first 10 points of MSD(τ) for fitting to Equation (2).

#### Construction of trafficking activity maps

Trafficking activity maps were constructed with ORIGIN software (RRID:SCR_014212, version 8.6 or above). First, we manually divided the cytoplasmic regions into 5 equally spaced regions, counted the fractions of the molecules and then calculated the mean diffusion coefficient within each region. We set a simplified model cell as a circle of which the central coordinate was x = y = 0 having radii $\left(r=\sqrt{x^{2}+y^{2}}\right)$ of 13 and 3 for the plasma and the nuclear membranes, respectively, and defined r = 4, 6, 8, 10 and 12 as the 5 equally spaced regions. We then created a 3D color map surface by plotting fractions of the molecules onto the z-axis of the model cell. For clarity, we represented one quarter (x ≥ 0, y ≥ 0) of the model cell. The surface colors representing the mean diffusion coefficient were determined by calculating the apparent fractions with diffusion coefficients of 0, 0.015 and 0.04 μm^2^/s by linear regression, and setting these values as thresholds.

### Quantification and statistical analysis

Data with error bars are the mean ± SEM unless otherwise indicated in the Figure Legends. The statistical significance of differences was assessed, as shown in the Figure legends, with ORIGIN or SPSS (RRID:SCR_019096, version 22). A p value less than 0.05 was considered to indicate a statistically significant difference.

## Data Availability

All data reported in this manuscript will be shared by the lead contact upon request. This report does not contain original code. Any additional information required to reanalyze the data reported herein is available from the lead contact upon request.
