# Supplementary material for: Three live-imaging techniques for comprehensively understanding the initial trigger for insulin-responsive intracellular GLUT4 trafficking
Source: iScience. 2022 Mar 26;25(4):104164. doi: 10.1016/j.isci.2022.104164 (PMC9010770; doi:10.1016/j.isci.2022.104164)
Supplement: Document S1. Figures S1–S4 [file mmc1.pdf]

iScience, Volume *25*

Supplemental information

Three live-imaging techniques for comprehensively understanding the initial trigger for insulin-responsive intracellular GLUT4 trafficking

Hiroyasu Hatakeyama, Ko Kobayashi, and Makoto Kanzaki

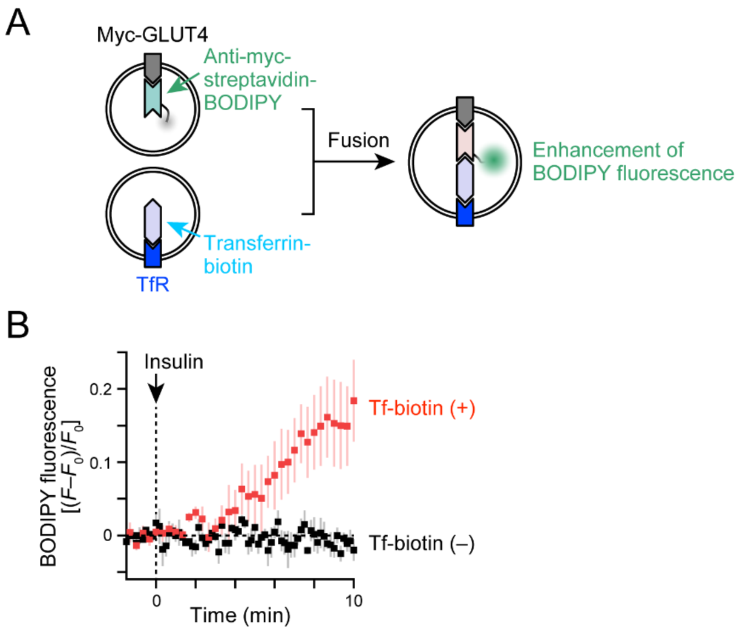

Figure S1. Requirement of TfR labeling with Tf-biotin in insulin-responsive increases in BODIPY fluorescence, related to Figure 1. (A) Schema for original fusion experiments in our previous study, which we traced only BODIPY fluorescence in the experiments. (B) Changes in BODIPY fluorescence in response to insulin stimulation (100 nM) at time 0 in 3T3L1 adipocytes. Myc-GLUT4 was labeled with BODIPY/streptavidin-conjugated antibodies, and after allowing internalization of labeled myc-GLUT4 we treated the cells with (red) or without (black) Tf-biotin. Data are the mean ± SEM (*n*=4).

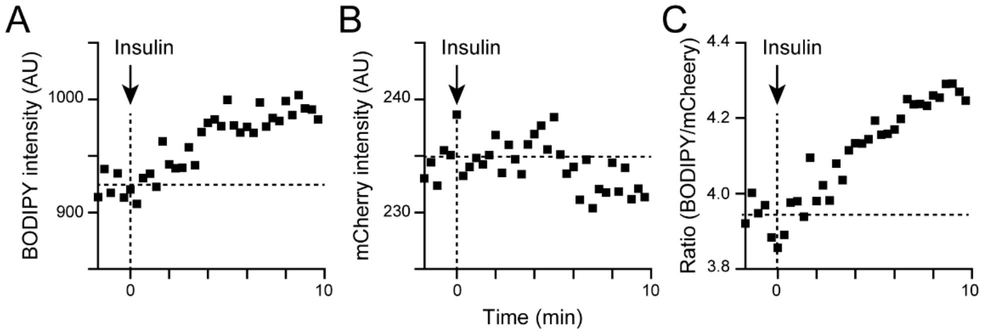

Figure S2. Insulin-responsive changes in the fluorescence, related to Figure 1. Changes in BODIPY fluorescence (A), mCherry fluorescence (B) and the BODIPY/mCherry ratio (C) in response to insulin stimulation (100 nM) at time 0 in the cell shown in Figure 1B.

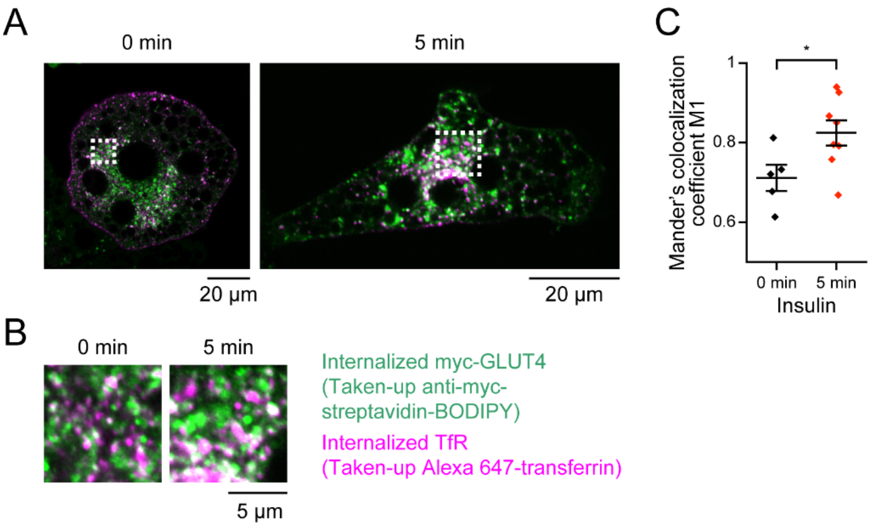

Figure S3. Brief insulin stimulation increases colocalization of internalized myc-GLUT4 with internalized TfR in 3T3-L1 adipocytes expressing myc-GLUT4-mCherry and TfR, related to Figure 1. (A) Fluorescent images of internalized myc-GLUT4 (green) and TfR (magenta) before (left) and after 5 min of insulin stimulation (right, 100 nM). For labeling, cells were first treated with BODIPY/streptavidin-conjugated anti-myc antibodies (biotin-loaded) for 1 h in the presence of 1 nM of insulin, washed for 3 h, treated with Alexa 647-conjugated transferrin for 5 min, and then washed for 10 min. After treatment without or with insulin for 5 min, cells were fixed, and the fluorescence was acquired. Scale bars = 20 µm. (B) Magnified images of boxed region in (A). (C) Changes in Mander's colocalization coefficient M1 of GLUT4 with TfR, which represents the fraction of GLUT4 in compartments containing TfR, after 5 min of insulin stimulation (100 nM). Solid lines represent the mean ± SEM. *p<0.05 by Welch's t-test.

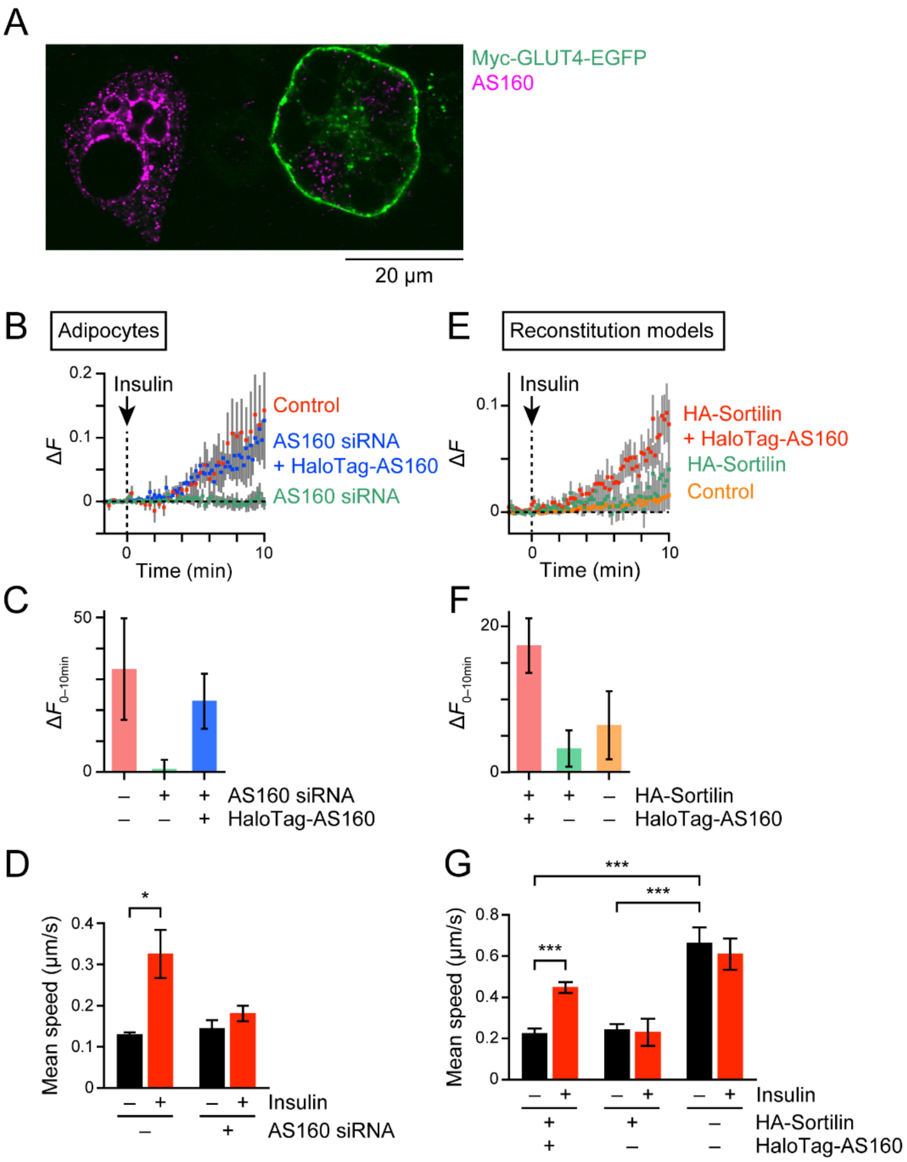

Figure S4. AS160-dependent heterotypic endosomal fusion and GLUT4 movement in 3T3L1 cells, related to figure 2. (A) Immunofluorescence of AS160 in 3T3L1 adipocytes simultaneously electroporated with both myc-GLUT4-EGFP and AS160 siRNA. Depletion of AS160 (magenta) was obviously observed only in the cells expressing myc-GLUT4-EGFP (green). (B–G) AS160-dependent heterotypic fusion and GLUT4 movement in 3T3L1 adipocytes expressing myc-GLUT4-mCherry and human transferrin receptor electroporating with or without AS160 siRNA and HaloTag-AS160 (B–D) or in 3T3L1 fibroblasts expressing only myc-GLUT4-mCherry, myc-GLUT4-mCherry + HA-sortilin, or myc-GLUT4-mCherry + HA-sortilin + HaloTag-AS160 (i.e., reconstitution models) (E–G). Changes in Δ*F* (B and E), area-under-curves of the Δ*F* during the 10-min period after insulin stimulation ($\Delta F_{0\text{–}10min}$) (C and F) and mean speeds of intracellular GLUT4 movements before and after insulin stimulation in the cells (*n*=3–8 cells). Data with error bars are the mean ± SEM. *p<0.05, ***p<0.001 by Tukey's multiple comparison test.
